# Robust enhanced leaf model implementation for beam‐matched linear accelerators: Impact of physical leaf gap variations

**DOI:** 10.1002/acm2.70895

**Published:** 2026-10-30

**Authors:** Soyoung Lee, Paulina E. Galavis, Siyoung Jang

**Affiliations:** ^1^ Department of Radiation Oncology NYU Langone Health New York New York USA; ^2^ Department of Radiation Oncology Boston Medical Center One Boston Medical Center Place Boston University Chobanian & Avedisian School of Medicine Boston Massachusetts USA

**Keywords:** dosimetric leaf gap, enhanced leaf model, leaf gap, MLC Modeling

## Abstract

**Background:**

Leaf‐gap variability may influence MLC modeling when implementing the Enhanced Leaf Model (ELM) in Eclipse v18.0 for beam‐matched linear accelerators (linacs) previously commissioned using legacy dosimetric leaf gap (DLG)–based models.

**Purpose:**

To characterize inter‐machine variability in calibrated leaf gaps and associated ELM‐derived MLC parameters across beam‐matched linacs and to develop a practical strategy for robust multi‐linac ELM implementation.

**Methods:**

Five TrueBeam linacs equipped with Millennium 120 MLC were grouped into two clinical sites (Group A: three linacs; Group B: two linacs) sharing a matched beam model within each group. Variations in calibrated leaf‐gap, *measured* DLG, and DLG (ELM) values were evaluated. Leaf‐gap variation was harmonized by adjusting *Gap* value in the machine setting while maintaining a machine‐averaged ELM across beam‐matched linacs. ELM performance was evaluated using three MLC test plans: dynamic MLC (dMLC), narrow MLC (nMLC), and static MLC (sMLC). Following *Gap* adjustment, DLG was remeasured, and DLG (ELM) values were verified. Central doses of dMLC fields were measured using an ionization chamber and compared with ELM‐calculated doses. Dose profiles were measured with radiochromic film, and were compared with calculated profiles before and after *Gap* adjustment. Profile agreement was further quantified using full width at half maximum (FWHM) and one‐dimensional gamma analysis (2%/1 mm, 10% dose threshold).

**Results:**

In Group A, ELM‐derived MLC parameters showed minimal variation, with DLG (ELM) values agreeing within 0.02 cm among the linacs with *Gap* values set to zero for all linacs. Film measurements demonstrated excellent agreement between calculated and measured dose distributions for all MLC test fields, with nMLC gamma passing rates generally exceeding 98% and FWHM differences within 0.2 mm. In Group B, an initial 0.05 cm difference in *Gap* value resulted in substantial discrepancies in dMLC tests, including central dose deviations of −7.5% and +5.0% relative to the corresponding calculated doses and approximately 0.05 cm difference in the measured dMLC field width. Following *Gap* adjustment, ELM‐derived parameters were converged and dosimetric agreement was improved markedly, with dMLC central dose differences reduced to within ± 1.1%. nMLC tests resulted in gamma pass rates exceeding 98%, and FWHM differences within 0.2 mm across all positions.

**Conclusions:**

The calibrated leaf‐gap, represented by the *Gap* value in the machine setting, directly influences DLG (ELM), *measured* DLG and dMLC dose response. Dynamic sweeping‐gap measurements provide a sensitive and efficient method for validating ELM performance and identifying residual inter‐machine discrepancies. A hybrid strategy combining machine‐averaged ELM parameters with harmonized leaf‐gap settings provides a practical and robust approach for multi‐linac ELM implementation.

## 1 INTRODUCTION

Modern radiotherapy increasingly relies on highly conformal delivery techniques such as intensity‐modulated radiotherapy (IMRT), volumetric modulated arc therapy (VMAT), and linac‐based stereotactic radiotherapy (SRT).^1^, ^2^ The accuracy of these techniques is closely linked to the precision of beam shaping and delivery, as they rely heavily on multileaf collimator (MLC)‐defined fields and dynamic MLC motion.^2^, ^3^, ^4^ Suboptimal MLC parameters in the treatment planning system (TPS) can lead to poor dosimetric performance in IMRT and VMAT.^5^, ^6^ This dependence can be particularly critical for small and off‐axis targets in SRT, where dosimetric and geometric uncertainties may translate into clinically meaningful dose deviations.^7^, ^8^ As treatment delivery expands across multiple beam‐matched linacs, maintaining dosimetric consistency and delivery interchangeability has become essential for robust clinical workflows and patient safety.

The Enhanced Leaf Model (ELM), introduced in Eclipse TPS v18.0 or higher (Varian Medical Systems, Palo Alto, CA, USA), improves the geometric representation of MLC leaf bodies and rounded leaf ends using divergent ray tracing.^7^ Several studies have reported improved agreement between measured and calculated dose distributions using the ELM compared with legacy dosimetric leaf gap (DLG)‐based MLC models.^7^, ^8^, ^9^, ^10^ Van Esch et al. demonstrated substantial improvement in dose calculation accuracy with ELM using dedicated static and dynamic MLC tests, highlighting discrepancies between dose calculations and measurements observed with the legacy MLC model.^7^ Moktan et al. further confirmed the improved agreement in clinical plans involving dynamic MLC motion, particularly for single‐isocenter multitarget (SIMT).^9^ ELM parameters are derived from leaf transmission and sweeping‐gap measurements performed on the corresponding treatment unit and therefore reflect the underlying machine‐specific MLC calibration. Previous studies have primarily evaluated machine‐specific ELMs on individual treatment units and demonstrated that ELM parameters correlate with the underlying MLC characteristics of the measured linac.^7^, ^9^ However, in multi‐linac environments, inter‐machine differences in calibrated leaf‐gap, as specified by the machine *Gap* value, could potentially propagate into the derived MLC modeling parameters and compromise beam‐matching consistency.

Recently, clinical adoption of the ELM has increased following upgrades to newer versions of Aria/Eclipse. A large cohort survey conducted by the Imaging and Radiation Oncology Core (IROC) Houston reported substantial variability in TPS MLC modeling parameters, particularly DLG.^11^ Such variability may partly originate from differences in leaf‐gap calibration and the resulting physical leaf geometry. While several studies have emphasized the importance of DLG in MLC modeling for accurate dose calculation,^12^, ^13^, ^14^, ^15^ the potential contribution of inherent physical leaf gap variability has not been systematically investigated. Furthermore, the relationship between the MLC *Gap* value, which is associated with actual physical leaf gaps, and derived MLC modeling parameters across beam‐matched linacs has not been well characterized. Consequently, the dosimetric consequences of submillimeter physical leaf gap variability remain unclear, especially for narrow dynamic MLC apertures commonly used in IMRT and VMAT.

The purpose of this study was to quantify the dependence of ELM‐derived MLC parameters on calibrated *Gap* setting across multiple beam‐matched linacs and to evaluate the resulting dosimetric impact of inter‐machine variability. In addition, we investigated a practical approach using machine‐averaged ELM parameters with converged *Gap* values to maintain robust and interchangeable delivery in a multi‐linac environment.

## 2 MATERIALS AND METHODS

Five Varian TrueBeams (TB) linacs equipped with Millennium 120 MLC were included in this study. The linacs were grouped into two clinical sites: three linacs in Group A and two linacs in Group B. Within each group, the linacs were dosimetrically equivalent and shared an identical beam model during commissioning in the Eclipse TPS v15.6. The MLC parameters in the legacy DLG model were empirically determined to produce optimal agreement between calculations and measurements for the given clinical practice. The *measured* DLG for each machine was obtained through linear extrapolation to zero dose using measurements from multiple sweeping gap tests,^4^ and has remained stable since commissioning.

Prior to ELM implementation in the Eclipse v18.0 clinical production, an Eclipse v18.0 test environment was initially commissioned for non‐MLC beams, followed by ELM configuration. Agreement between v15.6 and v18.0 was verified by recalculating the originally commissioned non‐MLC beam dataset, including Percent Depth Doses, output factors, and reference point dose calculations, with all calculated values agreeing within 1% prior to ELM MLC configuration. All MLC test plans were generated in the v18.0 test environment, then exported to the clinical production for ELM validation.

### 2.1 ELM parameters

Within the ELM framework, the LT parameter represents average attenuation through the leaf body including the drive screw cutout while DLG, hereafter referred to as DLG (ELM), is iteratively optimized from sweeping‐gap measurements.^7^ The ELM MLC parameters were derived from standardized MLC measurements provided by the manufacturer.^16^ The MLC measurement set consisted of one open field, two transmission fields blocked by MLC bank A and bank B, and three sweeping‐gap fields with gap widths of 4, 6, and 20 mm, which were delivered in a 10 × 10 cm^2^ collimated field. All measurements were performed using a 0.6 cm^3^ ionization chamber (PTW30013, PTW GmbH, Freiburg Germany) positioned at a depth of 10 cm in a solid water phantom with a source‐to‐surface distance (SSD) of 90 cm. For each linac, MLC calibration/initialization was performed in Service mode prior to the measurements. Machine‐specific ELM parameters were derived independently for each machine. For each group, a machine‐averaged ELM was generated by entering averaged measurements and subsequently implemented in the TPS. Table S‐1 summarizes the MLC parameters defined for the MLC models in each TPS version and the MLC measurements required for the parameter.

### 2.2 Gap value adjustment

The actual physical leaf gap between opposing closed MLC leaves can be mechanically measured during linac commissioning to ensure beam‐matching consistency across linacs. On the TrueBeam platform, the physical leaf gaps are calibrated and specified by a single *Gap* value under MLC Initialization with intermediate rights in Service mode at the treatment console. Since both *measured* DLG and DLG (ELM) values are primarily governed by the calibrated leaf‐gap configured for an individual treatment unit, adjustment of *Gap* value was considered when inter‐machine *Gap* values in the machine setting were different. Although *Gap* adjustment requires comprehensive re‐validation, standardizing the *Gap* value across the machines could reduce inter‐machine dosimetric variability while preserving a single ELM and maintaining delivery consistency. In this study, the *Gap* adjustment was performed by distributing the inter‐machine difference in *measured* DLG between the two machines.

Following adjustment, the MLC measurements were repeated to verify the consistency of *measured* DLG and agreement of DLG (ELM) values with the machine‐averaged ELM. A 3‐mm dynamic sweeping‐gap field was used to assess the gap‐dependent integral dose response through central‐dose measurements. The dose was measured using an ionization chamber. Adjustment was performed using 6 MV photon beams and was subsequently verified for all other clinically available photon energies.

### 2.3 Validation of ELM

ELM validation was performed on each linac using three representative MLC test patterns adapted from previously described methodologies^7^:

1. Dynamic MLC field (dMLC): A 2‐cm field composed of overlapping 3‐mm sweeping gaps was delivered at the beam central‐axis (CAX), at 4 and 8 cm off‐axis (OAX) positions. A slight overlap was incorporated to produce a smooth composite profile. Note that different degrees of overlap between the 3 mm sweeping gaps were applied for Group A and Group B, accounting for their inherent physical leaf gap differences into the earlier version of MLC model in each TPS.
2. Narrow MLC field (nMLC): A 3‐mm MLC slit delivered at CAX, at 5 and 10 cm OAX to evaluate penumbra and small‐aperture sensitivity to leaf gap variation. Agreement between calculated and measured dose profiles was quantified using full width at half maximum (FWHM) and one‐dimensional (1D) gamma analysis (2%/1 mm criteria, 10% dose threshold).
3. Static MLC field (sMLC): Four sequential 4 × 20 cm^2^ MLC segments were delivered with intentional overlap between adjacent subfields to enhance sensitivity to leaf gap variation and to evaluate dosimetric impacts at junctions associated with rounded leaf ends.

All plans were calculated for 6 MV using the Anisotropic Analytical Algorithm with 1‐mm grid resolution. Dose profiles were measured with radiochromic films (GafChromic EBT4, Ashland Inc., Covington, KY) placed at 10 cm depth in solid water at 90 cm SSD. The irradiated films were scanned 24 hours post‐irradiation using a flatbed scanner (EPSON 10000XL, Seiko Epson Corp., Japan) at 300 dpi and 24‐bit color, and dose conversion was performed using the red channel with same‐batch calibration. Absolute dose verification for sMLC and dMLC fields was cross‐checked with ionization chamber measurements using an Exradin A1SL chamber (Standard Imaging, Middleton, WI).

## 3 RESULTS

### 3.1 MLC parameter variability

Table 1 shows the MLC parameters obtained with 6 MV for each linac and MLC models configured in Eclipse TPS v15.6 and v18.0. For the Group A linacs, the MLCs were commissioned with an identical *Gap* value of 0.0 cm. The *measured* DLG values showed good inter‐machine consistency, with a maximum difference of 0.012 cm. The machine‐specific DLG (ELM) values followed a similar trend. The *ELM DLG model* was derived from the averaged MLC measurements in the TPS. For the Group B linacs, the MLCs were initially configured with a 0.05 cm difference in the *Gap* value, resulting in differences of approximately 0.06 cm in both *measured* DLG and DLG (ELM). Following adjustment of the *Gap* value, the *measured* DLG values converged to 0.126 cm for TB1B and 0.132 cm for TB2B, and DLG (ELM) values were −0.054 cm and −0.051 cm, respectively. The *ELM DLG model* was derived from the averaged initial MLC measurements before *Gap* adjustment. After *Gap* adjustment using 6 MV, good agreement was also achieved across the other available energies for both the *measured* DLG and the corresponding DLG (ELM) values, although the *Gap* adjustment was performed using a single energy. The MLC parameters for all other energies available at each linac are provided in Table S‐2.

**TABLE 1:** MLC parameters for each linac.

Group A				
Linac#	LT	*Gap* (cm)	*measured* DLG (cm)	DLG (ELM) (cm)
TB2A	0.015	0.00	0.096	−0.095
TB3A	0.015	0.00	0.103	−0.081
TB4A	0.014	0.00	0.108	−0.076
*ELM DLG model*	*0.015*			*−0.083*
*Legacy DLG model*	*0.015*		*0.125*	

Group B							
		Before adjustment			After adjustment		
Linac#	LT	*Gap* (cm)	*measured* DLG (cm)	DLG (ELM) (cm)	*Gap* (cm)	*measured* DLG (cm)	DLG (ELM) (cm)
TB1B	0.013	0.00	0.103	−0.079	0.026	0.126	−0.054
TB2B	0.013	0.05	0.163	−0.020	0.020	0.132	−0.051
*ELM DLG model*	*0.013*			*−0.050*			
*Legacy DLG model*	*0.014*		*0.130*				

*Note*: The LT and DLG (ELM) values for each machine were derived from individual MLC measurements for ELM configuration in v18.0 TPS. The *ELM DLG model* was derived from averaged MLC measurements. The *legacy DLG model* reports empirically determined LT and legacy DLG values in Eclipse v15.6 TPS.

Abbreviations: DLG, dosimetric leaf gap; ELM, enhanced leaf model; LT, leaf transmission.

Figure 1a demonstrates a strong linear relationship between the *Gap* value and the corresponding *measured* DLG (R^2^ = 0.95). The differences in the *measured* DLGs between linacs were systematically reduced through *Gap* adjustment. Small differences in *measured* DLGs remained among the Group A linacs configured with the same *Gap* value, reflecting variability in the actual physical leaf gaps. Figure 1b demonstrates a strong linearity between *measured* DLG and the corresponding DLG (ELM) (R^2^ = 0.99), indicating that the convergence of the DLG (ELM) values can be achieved by matching the *measured* DLGs values.

**FIGURE 1:**
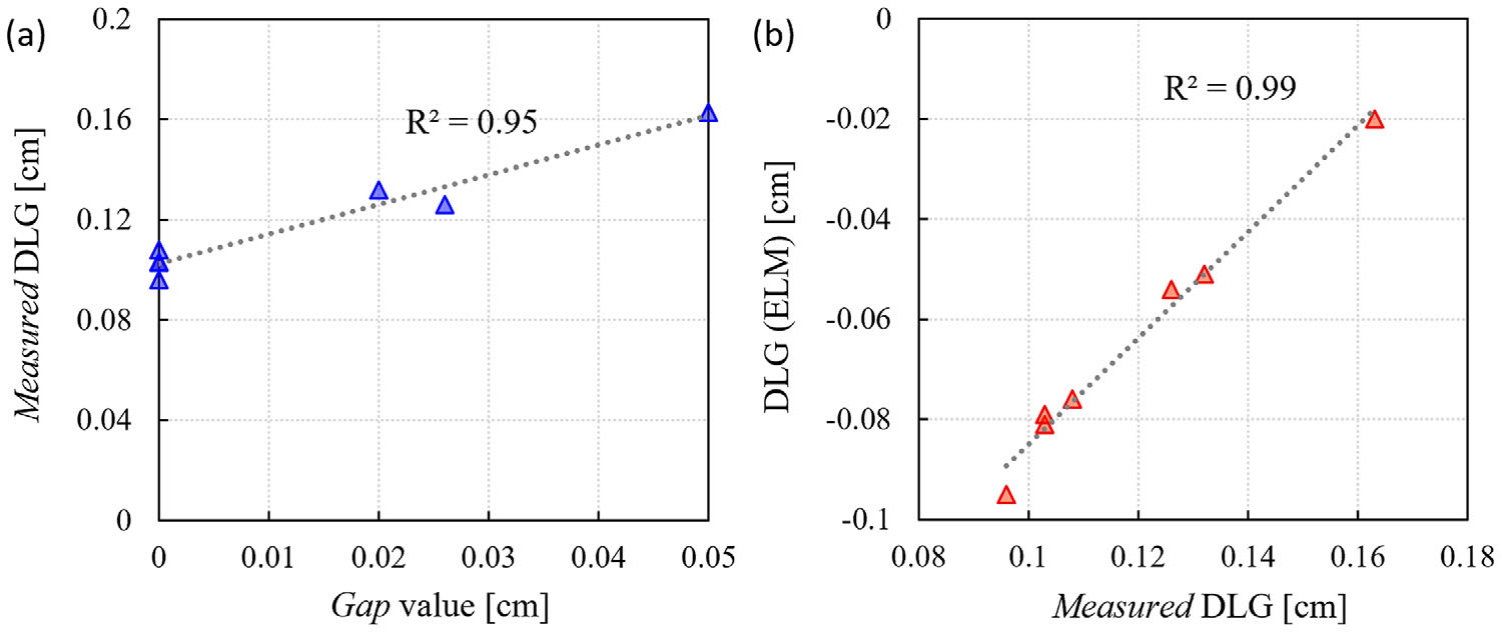
Relationships among the MLC *Gap* value, *measured* DLG, and DLG (ELM) for the five linacs. (a) Relationship between the *Gap* value and the corresponding *measured* DLG. (b) Relationship between DLG (ELM) and *measured* DLG.

Following verification of the *measured* DLG and DLG (ELM) values, chamber measurements were acquired at the center of each dMLC field and compared with calculated doses as the dMLC test fields demonstrated the greatest sensitivity to inter‐machine leaf‐gap variation. In Group A, the chamber‐measured central doses differed from the calculated doses by −1.4%, 2.3%, and 2.8% at CAX for TB2A, TB3A, and TB4A, respectively. In Group B, a 0.05 cm difference in *Gap* between the two linacs resulted in substantial deviations between measured and calculated central doses. The adjustment of the *Gap* from 0 to 0.026 cm reduced the CAX dose differences from −7.5% to −1.1% for TB1B, while adjustment from 0.05 to 0.02 cm reduced the difference from 5.0% to −0.2% for TB2B. Similar Gap‐dependent behavior was observed at off‐axis positions (4 and 8 cm OAX).

Figure 2 shows the measured dMLC dose profiles on EBT4 compared with the calculated dose profiles. Each dMLC field was calculated and measured individually at each position to minimize the effect of the scatter contributions to the field within the solid water phantom, particularly for the off‐axis measurements. The measured profiles at Group A linacs showed good agreement with the calculated profiles, with FWHM differences within ± 0.3 mm across all evaluated positions. In Group B, the measured profiles showed significant deviations from the calculated profiles. For TB1B, the smaller DLG (ELM) value (= −0.079 cm) resulted in a narrower effective aperture and reduced measured dose relative to the calculated profile. Before *Gap* adjustment, larger differences were observed, particularly for TB1B, with FWHM differences ranging from −0.64 to −0.46 mm, while those for TB2B were within 0.2 mm for TB2B. Following *Gap* adjustment, FWHM differences were within ± 0.2 mm for both linacs across all evaluated positions. The dose difference in the dMLC profiles between Groups A and B reflects the different degrees of overlap between the 3‐mm sweeping gaps used to generate the dMLC fields, as described in the Methods.

**FIGURE 2:**
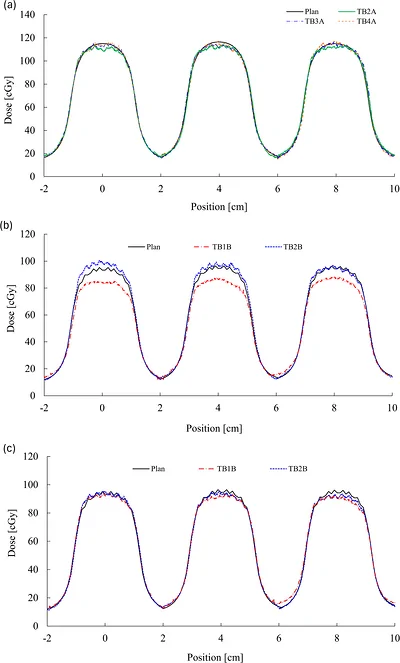
Measured and calculated dose profile comparisons along the leaf motion direction for dMLC fields: (a) Group A, and Group B (b) before and (c) after *Gap* adjustment.

Figure 3 shows the calculated and measured nMLC dose profiles for Group A Linacs. For Group A, each nMLC field was individually calculated and measured at CAX, 5 cm, and 10 cm OAX. Overall, the calculated dose profiles with the clinical ELM demonstrated excellent agreement with film measurements across all linacs and positions as shown in Figure 3. Minor differences were observed in the low‐dose region, which was expected due to increased uncertainty in film measurements at lower dose levels. All measured profiles resulted in gamma passing rates (2%/1 mm, 10% threshold) of 98%–100%, with most profiles achieving 100% passing rates. FWHM derived from measured and calculated profiles were highly consistent, resulting in differences below 0.2 mm.

**FIGURE 3:**
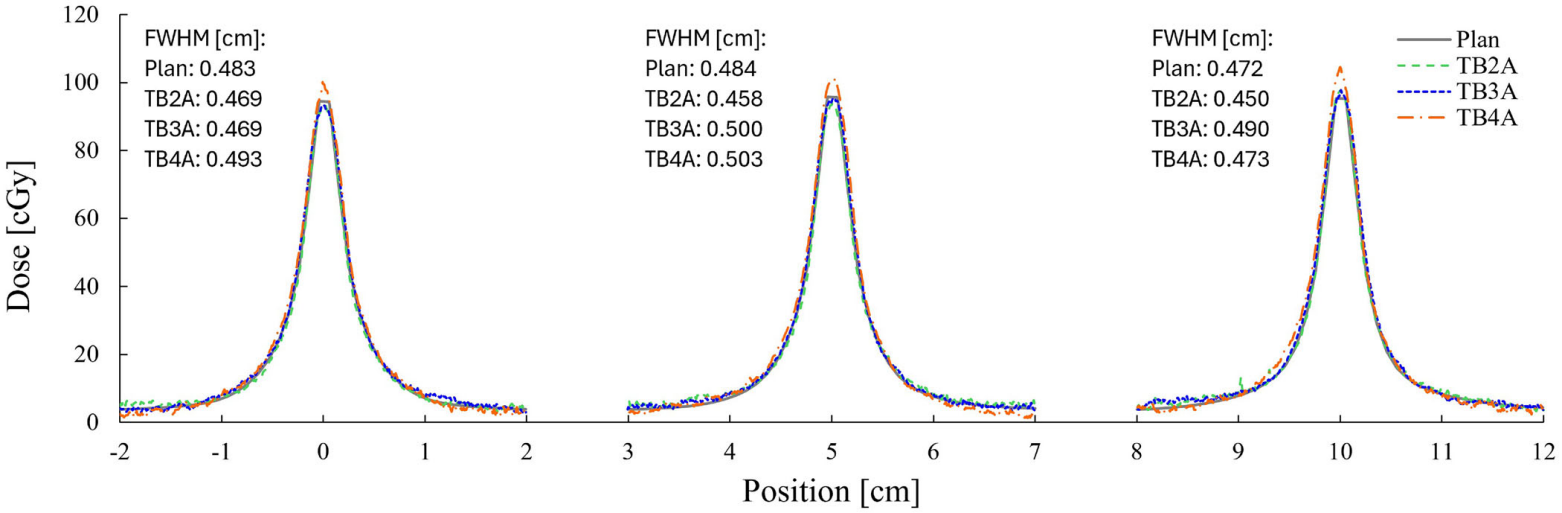
Comparison between planned and measured nMLC dose profiles at CAX, 5 cm, and 10 cm OAX for Group A. For each axis, nMLC plan was calculated using the ELM DLG model and delivered individually onto film positioned at the center of the water phantom. FWHM values are shown for both the planned and measured dose profiles.

Figure 4 shows the calculated and measured nMLC dose profiles for Group B Linacs. In Group B, nMLC fields at CAX, 5 cm, and 10 cm OAX were sequentially delivered onto a single film. Note that the calculated plan profiles remained unchanged before and after *Gap* adjustment, reflecting use of the same clinical ELM. Prior to adjustment, TB1B showed noticeable reduced peak dose and narrower aperture in the measured dose profiles due to the smaller leaf gap relative to the calculated dose profile. Despite the difference between its machine‐specific DLG (ELM) and the machine‐averaged clinical DLG, TB2B remained in close agreement with the calculated profiles before adjustment because of its *Gap* value setting of 0.05 cm. In contrast, TB1B exhibited larger dose deviations, suggesting increased dosimetric sensitivity associated with smaller *Gap* value settings. Gamma passing rates were originally 95.3%, 95.7%, and 96.0% for TB1B, and 100.0%, 99.0%, and 99.8% for TB2B at CAX, 5 and 10 cm OAX, respectively. Following *Gap* adjustment, measured dose profile agreement improved particularly for TB1B across all positions. The gamma passing rates improved to 100.0%, 100.0%, and 98.3% for TB1B, and remained similar at 99.8%, 100.0%, and 99.78% for TB2B at CAX, 5 and 10 cm OAX, respectively. FWHM differences were correspondingly reduced to below 0.02 cm. These results demonstrate that *Gap* harmonization improved agreement between measured and calculated profiles while maintaining consistency with the machine‐averaged clinical ELM.

**FIGURE 4:**
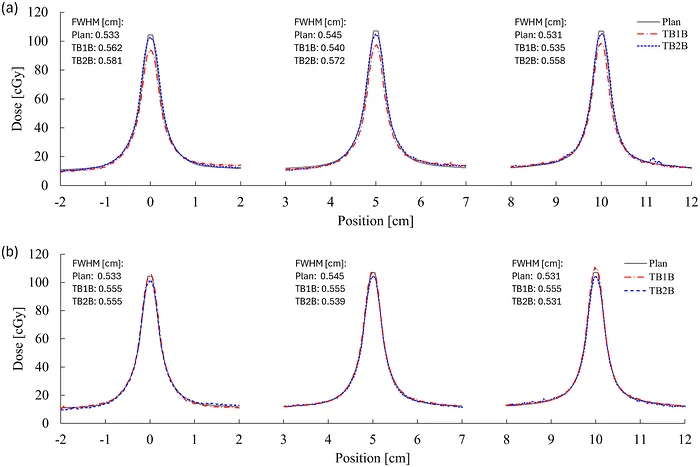
Comparison between plan and measured nMLC dose profiles at CAX, 5 cm, and 10 cm OAX for Group B: (a) before and (b) after *Gap* adjustment. The calculated profiles were extracted from the composite nMLC plan at each axis. Measurements were acquired on a single film by sequential delivery of all nMLC fields.

Figure 5 compares the measured and ELM‐calculated sMLC profiles over ± 2 cm from the central axis. Independent ionization chamber measurements at ± 2 cm from CAX confirmed the film‐based absolute dose agreement within 0.5%. In Group A, calculated and measured profiles demonstrated good overall agreement, including at the leaf‐junction regions (Figure 5a). Gamma passing rates were 95.5%, 94.3%, and 95.9% for TB2A, TB3A, and TB4A, respectively. In Group B, profile discrepancies were observed at the junction regions before *Gap* adjustment (Figure 5b,c). Gamma passing rates before and after *Gap* adjustment were 99.7% and 98.8% for TB1B, and 91.4% and 98.7% for TB2B, respectively. Following *Gap* adjustment, the gamma passing rate remained comparable for TB1B and improved for TB2B. The mean central‐dose difference relative to the calculated profile was 4.1%, 2.0%, and 3.0% for TB2A, TB3A, and TB4A, respectively. The mean central‐dose difference was 0.86% for TB1B and 9.83% for TB2B. Following *Gap* adjustment, agreement improved, with mean central‐dose differences of 2.97% and 0.77% for TB1B and TB2B, respectively.

**FIGURE 5:**
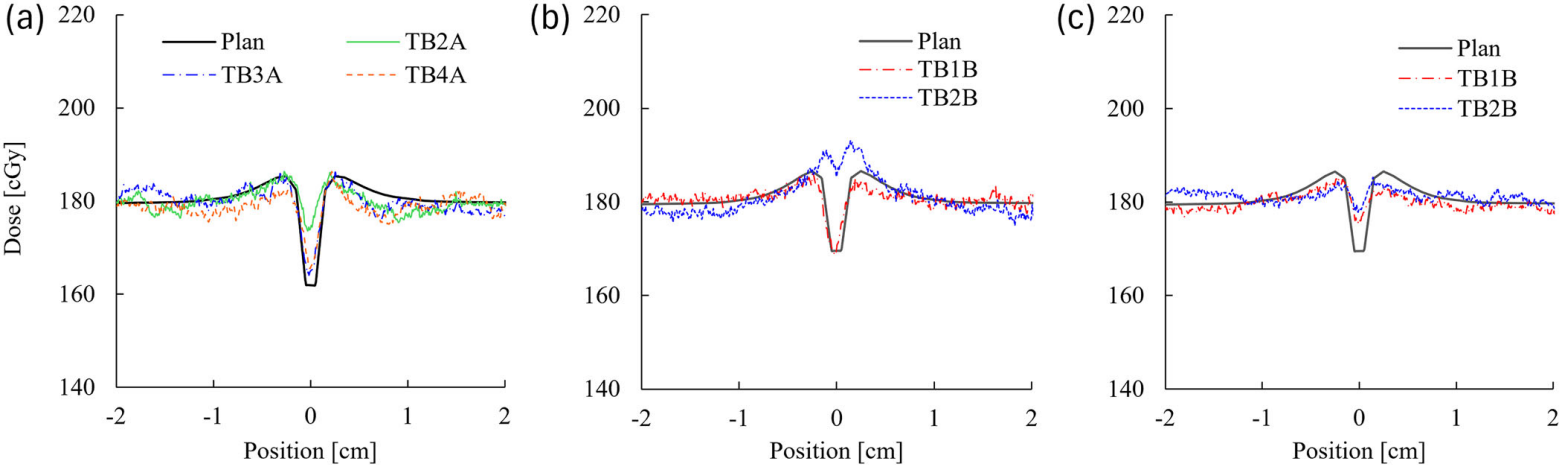
Comparison of measured and calculated sMLC dose profiles at CAX: (a) Group A, (b) Group B before (c) and after *Gap* adjustment. Profiles are displayed over ± 2 cm at the central axis to highlight the junction dose differences from sequential segment delivery.

## 4 DISCUSSION

In ELM, MLC characteristics are defined by leaf gap and positional accuracy of individual leaves governed by MLC motor driving‐force. Recent studies have explored the feasibility of implementing a single machine‐averaged ELM across beam‐matched linacs. The studies indicated that the machine‐averaged ELMs resulted in negligible clinical impact, but performed only within well‐matched linacs.^17^, ^18^ Ruggieri et al. found better agreement in a SIMT case when a machine‐specific ELM was used for a single treatment unit.^2^ In the present study, we systematically investigated the direct relationship variation of *Gap* value versus *measured* DLG and derived DLG (ELM), which showed that measured MLC parameters were strongly dependent on the calibrated leaf gap of each treatment unit. Additionally, inter‐machine variations in both mechanical and dosimetric characteristics were explicitly quantified, and their dosimetric impact was evaluated using a range of clinically relevant MLC test plans.

The study shows that harmonizing *Gap* values among linacs provides a robust approach for multi‐linac ELM modeling, which demonstrates that empirical tuning of MLC parameters alone does not improve MLC performance across beam‐matched linacs. Linear relationship among *Gap* value, *measured* DLG, and DLG (ELM) confirmed the significant coupling between *Gap* value in machine setting and MLC modeling parameters in the TPS. However, this analysis was based on only five treatment units, and greater DLG variability may be observed across a larger population of linacs.^19^ These findings provide a potential explanation for the variability in MLC modeling parameters observed in multi‐institutional surveys of clinical beam models.^12^ For TB1B and TB2B linacs, a legacy DLG value of 0.130 cm was used in the TPS v15.6 to match the two linacs dosimetrically, which was the average value of *measured* DLGs of two machines rather than utilizing the same *Gap* value in the machine settings. However, in the TPS v18.0, the *Gap* value of each machine was adjusted by 0.02 cm to mitigate potential MLC collision risk of setting the *Gap* value of zero, and to minimize clinical impacts on the dosimetric change in the calculated treatment plans during the transition from the legacy DLG‐based model in v15.6 to the ELM in v18.0. Based on these findings, for well‐matched linacs with the *Gap* value set to 0, the present results suggest that inter‐machine differences within approximately 0.02 cm in DLG (ELM), *measured* DLG values of approximately 0.01 cm, and dMLC central‐dose deviations within ± 3%, may provide practical criteria for evaluating consistency with a machine‐averaged ELM. These values should not be considered universal tolerance limits as the appropriate *Gap* adjustment for harmonizing linacs may be clinic‐specific and should account for the existing MLC calibration and DLG values from the previous model to maintain dosimetric consistency when transitioning between MLC models.

Among the MLC test fields, the dMLC field demonstrated particularly high sensitivity to gap variability. Unlike the nMLC and sMLC tests, the dMLC test detected integral dose changes resulting from gap‐dependent leaf‐overlap during dynamic sweeping‐gap motion. In our study, *Gap* adjustment of approximately 0.25–0.30 mm reduced the central dose differences in the dMLC fields by approximately 5%–6%. Smaller leaf gaps produced a disproportionately larger reduction in dMLC dose response, indicating increased sensitivity of narrow dynamic apertures to leaf‐overlap effects. These variations may propagate into highly modulated treatments using numerous small dynamic apertures.

Leveraging these observations, we established a practical validation workflow to implement ELM for matched machines. First, standardized MLC measurements were repeated to confirm that the *measured* DLG and derived DLG (ELM) values were consistent with the machine‐averaged ELM parameters. Second, the central dose from the dMLC delivery was measured using an ionization chamber to verify dose agreement across linacs. This provides immediate confirmation of dose consistency and identification of residual gap mismatch across the machines, whereas film‐based validation requires longer processing and analysis time. Nevertheless, MLC verification with high‐resolution dosimetry remains necessary for nMLC, sMLC and dMLC tests, to confirm beam penumbra agreement at low‐dose regions, the dosimetric behavior of leaf‐end junctions, and dose profiles of dynamic fields generated by leaf‐end modeling, since these effects could influence dose distributions in clinical plans. Thus, the proposed validation strategy provides a practical and time‐efficient method for confirming ELM implementation following *Gap* adjustment if applicable.

To complete the clinical implementation of ELM, several patient‐specific QAs were performed for VMAT and IMRT plans. However, conventional patient‐specific QA may not be sufficiently sensitive to detect subtle MLC‐modeling differences.^20^ As the primary focus of this study was to establish robust ELM validation using dedicated MLC test plans, comprehensive evaluation of ELM performance across all patient‐specific QA scenarios was beyond the scope of this work.

Several limitations warrant consideration. First, the study focused on a single MLC platform using only 6 MV photon beams for ELM implementation. Second, validation fields were designed to isolate the sensitivity of MLC modeling and may not fully represent the complexity of clinical plans. However, the linear relationship between *Gap* values and derived modeling parameters suggests that the observed behavior is intrinsic to the modeling framework rather than plan‐specific. Finally, although off‐axis evaluations demonstrated similar trends, slightly reduced agreement may reflect limitations associated with film‐based measurements. However, validation within 10 cm off‐axis from the central axis remains representative for most clinical treatment scenarios.

## 5 CONCLUSION

Sub‐millimeter differences in calibrated leaf gap can produce clinically meaningful variations in dynamic MLC dose response. Prior harmonization of *Gap* value before implementing machine‐averaged ELM parameters provides a practical and clinically robust approach for maintaining delivery consistency across beam‐matched linacs while enabling efficient ELM implementation. Furthermore, dynamic sweeping‐gap measurements offer a sensitive and time‐efficient method for validating ELM performance, such that residual inter‐machine discrepancies can not only be identified but also compensated for by fine‐tuning the *Gap* value in machine service mode. These findings provide practical guidance for institutions seeking to deploy ELM across multiple linacs while preserving clinical workflow, delivery interchangeability, and dosimetric consistency.

## AUTHOR CONTRIBUTIONS

Soyoung Lee wrote the original manuscript and acquired and analyzed data. Paulina E. Galavis reviewed and edited the manuscript. Siyoung Jang analyzed the data and reviewed and edited the manuscript.

## CONFLICT OF INTEREST STATEMENT

The authors declare no conflicts of interest.

## ETHICAL APPROVAL

This study used phantom measurements and treatment planning system data for quality assurance and commissioning purposes. No human participants or identifiable patient data were involved. Therefore, institutional review board (IRB) approval and informed consent were not required under institutional policies.

## Supporting information



Supporting Information

## Data Availability

The data that support the findings of this study are available from the corresponding author upon reasonable request.

## References

[1] Palmer JD , Sebastian NT , Chu J , et al. Single‐isocenter multitarget stereotactic radiosurgery is safe and effective in the treatment of multiple brain metastases. Adv Radiat Oncol. 2019;5(1):70‐76. doi:10.1016/j.adro.2019.08.013 PMC7004936 32051892

[2] Ruggieri R , Naccarato S , Mazzola R , et al. Linac‐based radiosurgery for multiple brain metastases: comparison between two mono‐isocenter techniques with multiple non‐coplanar arcs. Radiother Oncol. 2019;132:70‐78. doi:10.1016/j.radonc.2018.11.014 30825972

[3] Saez J , Bar‐Deroma R , Bogaert E , et al. Universal evaluation of MLC models in treatment planning systems based on a common set of dynamic tests. Radiother Oncol. 2023;186:109775. doi:10.1016/j.radonc.2023.109775 37385376

[4] LoSasso T , Chui C , Ling C . Physical and dosimetric aspects of a multileaf collimation system used in the dynamic mode for implementing intensity modulated radiotherapy. Med Phys. 1998;25(10):1919‐1927. doi:10.1118/1.598381 9800699

[5] Glenn MC , Brooks F , Peterson CB , et al. Photon beam modeling variations predict errors in IMRT dosimetry audits. Radiother Oncol. 2022;166:8‐14. doi:10.1016/j.radonc.2021.10.021 PMC8863621 34748857

[6] Szpala S , Cao F , Kohli K . On using the dosimetric leaf gap to model the rounded leaf ends in VMAT/RapidArc plans. J Appl Clin Med Phys. 2014;15(2):4484. doi:10.1120/jacmp.v15i2.4484 PMC5875471 24710433

[7] Van Esch A , Kulmala A , Rochford R , et al. Testing of an enhanced leaf model for improved dose calculation in a commercial treatment planning system. Med Phys. 2022;49:7754‐7765. doi:10.1002/mp.16019 36190516

[8] Vieillevigne L , Khamphan C , Saez J , Hernandez V . On the need for tuning the dosimetric leaf gap for stereotactic treatment plans in the Eclipse treatment planning system. J Appl Clin Med Phys. 2019;20(7):68‐77. doi:10.1002/acm2.12656 PMC6612699 31225938

[9] Moktan H , Jiang H , Li HH , Guida K . Impact of enhanced leaf model on dose calculation accuracy in single‐isocenter multitarget stereotactic radiosurgery treatments. J Appl Clin Med Phys. 2025;26(6):e70039. doi:10.1002/acm2.70039 PMC12148782 39971711

[10] Yang Y , Shoniyozov K , Li T , et al. Evaluation of the accuracy of an enhanced MLC leaf model for Linac‐based stereotactic radiosurgery. J Appl Clin Med Phys. 2025;26(7):e70143. doi:10.1002/acm2.70143 PMC12256662 40653786

[11] Glenn MC , Peterson CB , Followill DS , et al. Reference dataset of users’ photon beam modeling parameters for the Eclipse, Pinnacle, and RayStation treatment planning systems. Med Phys. 2020;47:282‐288. doi:10.1002/mp.13892Digital Object Identifier (DOI) PMC6980266 31667870

[12] Koger B , Price R , Da Wang , et al. Impact of the MLC leaf‐tip model in a commercial TPS: dose calculation limitations and IROC‐H phantom failures. J Appl Clin Med Phys. 2020;21:82‐88. doi:10.1002/acm2.12819 PMC7021005 31961036

[13] Chauvet I , Petitfils A , Lehobey C , et al. The sliding slit test for dynamic IMRT: a useful tool for adjustment of MLC related parameters. Phys Med Biol. 2005;50(4):563‐580. doi:10.1088/0031-9155/50/4/001 15773620

[14] Van Esch A , Bohsung J , Sorvari P , et al. Acceptance tests and quality control (QC) procedures for the clinical implementation of intensity modulated radiotherapy (IMRT) using inverse planning and the sliding window technique: experience from five radiotherapy departments. Radiother Oncol. 2002;65(1):53‐70. doi:10.1016/s0167-8140(02)00174-3 12413675

[15] Wang X , Ma C , Zhang T , et al. Impact of two‐dimensional dosimetric leaf gap on SRS‐VMAT for single‐isocenter multi‐target brain metastases. Sci Rep. 2025;15(1):29627. doi:10.1038/s41598-025-14300-9 PMC12350949 40804092

[16] Varian Medical Systems, Inc . Enhanced leaf modeling measurement procedure, document ID P1062983‐002‐B. Varian Medical Systems, Inc., 2023.

[17] Panagi R , Caines R , Rowbottom CG . Dosimetric sensitivity of an enhanced leaf model (ELM) for individual versus averaged machines. J Appl Clin Med Phys. 2025;26:e14621. doi:10.1002/acm2.14621 PMC11969076 39902660

[18] Miyasaka R , Hirai R , Suda Y , et al. Multi‐institutional study on the commissioning and clinical implementation of the TrueBeam enhanced leaf model in the eclipse treatment planning system. J Appl Clin Med Phys. 2025;26(12):e70360. doi:10.1002/acm2.70360 PMC12646809 41290357

[19] Ghazal M , Södergren L , Westermark M , Söderström J , Pommer T . Dosimetric and mechanical equivalency of Varian TrueBeam linear accelerators. J Appl Clin Med Phys. 2020;21(12):43‐53. doi:10.1002/acm2.13058 PMC7769408 33070456

[20] Erickson B , Cui Y , Alber M , et al. Independent Monte Carlo dose calculation identifies single isocenter multi‐target radiosurgery targets most likely to fail pre‐treatment measurement. J Appl Clin Med Phys. 2024;25:e14290. doi:10.1002/acm2.14290 PMC11163499 38289874

