# Supplementary material for: Robust enhanced leaf model implementation for beam‐matched linear accelerators: Impact of physical leaf gap variations
Source: J Appl Clin Med Phys. 2026 Oct 30;27(11):e70895. doi: 10.1002/acm2.70895 (PMC13721898; doi:10.1002/acm2.70895)
Supplement: Supplementary file 1 [file ACM2-27-e70895-s001.docx]

Supplementary Materials

TABLE S-1 MLC parameters in TPS before and after v18.0.

| DLG-based MLC Parameters  (TPS v15.6) | ELM MLC Parameters  (TPS v18.0) | MLC Measurements |
| --- | --- | --- |
| - LT: represents transmission through the homogeneous, thin layer. | - LT: represents transmission through the leaf body including the drive screw cutout. | 1) Open 10 x 10 cm^2^ field,  2) transmission fields blocked by MLC bank A and bank B,  3) three sweeping-gap fields with gap widths of 4 mm, 6 mm, and 20 mm on a 10 x 10 cm^2^ field size. |
| - DLG (legacy): represents the zero offsets between opposing leaves in the TPS, accounting for the effect of the rounded leaf-tip.^4^ | - DLG (ELM): iteratively optimized from the sweeping-gap measurements.^17^ |  |

Abbreviations: ELM, enhanced leaf model; TPS, treatment planning system; LT, leaf transmission; DLG, dosimetric leaf gap.

Table S-2 MLC parameters for other energies available at each linac in Group A and B.

| Group A | | | | | |
| --- | --- | --- | --- | --- | --- |
| Linac# | LT | *Gap*  (cm) | *measured* DLG  (cm) | DLG (ELM)  (cm) |  |
| **6MV FFF** |  |  |  |  |  |
| TB2A | 0.013 | 0.00 | 0.094 | – 0.103 |  |
| TB3A | 0.013 | 0.00 | 0.107 | – 0.088 |  |
| TB4A | 0.012 | 0.00 | 0.110 | – 0.084 |  |
| *ELM DLG model* | *0.012* |  |  | *– 0.091* |  |
|  |  |  |  |  |  |
| **10 MV** |  |  |  |  |  |
| TB2A | N/A | 0.00 | N/A | N/A |  |
| TB3A | N/A | 0.00 | N/A | N/A |  |
| TB4A | 0.016 | 0.00 | 0.128 | – 0.066 |  |
| *ELM DLG model* | *0.016* |  |  | *– 0.066* |  |
|  |  |  |  |  |  |
| **10 MV FFF** |  |  |  |  |  |
| TB2A | 0.015 | 0.00 | 0.100 | – 0.090 |  |
| TB3A | 0.015 | 0.00 | 0.112 | – 0.078 |  |
| TB4A | 0.014 | 0.00 | 0.118 | – 0.071 |  |
| *ELM DLG model* | 0.015 |  |  | *–* 0.080 |  |
| **15 MV** |  |  |  |  |  |
| TB2A | 0.017 | 0.00 | 0.109 | – 0.085 |  |
| TB3A | 0.017 | 0.00 | 0.121 | – 0.072 |  |
| TB4A | 0.016 | 0.00 | 0.129 | – 0.064 |  |
| *ELM DLG model* | *0.016* |  |  | *– 0.074* |  |

| Group B |  |  | | |  | | |
| --- | --- | --- | --- | --- | --- | --- | --- |
|  |  | Before adjustment | | | After adjustment | | |
| Linac# | LT | *Gap*  (cm) | *measured* DLG  (cm) | DLG (ELM)  (cm) | *Gap*  (cm) | *measured* DLG (cm) | DLG (ELM)  (cm) |
| **6MV FFF** |  |  |  |  |  |  |  |
| TB1B | 0.011 | 0.00 | 0.088 | – 0.086 | 0.026 | 0.114 | – 0.062 |
| TB2B | 0.011 | 0.05 | 0.148 | – 0.026 | 0.020 | 0.117 | – 0.057 |
| *ELM DLG model* | *0.011* |  |  | *– 0.056* |  |  |  |
|  |  |  |  |  |  |  |  |
| **10 MV** |  |  |  |  |  |  |  |
| TB1B | 0.015 | 0.00 | 0.119 | – 0.071 | 0.026 | 0.143 | – 0.046 |
| TB2B | 0.015 | 0.05 | 0.176 | – 0.013 | 0.020 | 0.146 | – 0.043 |
| *ELM DLG model* | *0.015* |  |  | *– 0.042* |  |  |  |
|  |  |  |  |  |  |  |  |
| **10 MV FFF** |  |  |  |  |  |  |  |
| TB1B | 0.013 | 0.00 | 0.115 | – 0.071 | 0.026 | 0.134 | – 0.050 |
| TB2B | 0.013 | 0.05 | 0.169 | – 0.014 | 0.020 | 0.137 | – 0.046 |
| *ELM DLG model* | *0.013* |  |  | *– 0.042* |  |  |  |
|  |  |  |  |  |  |  |  |
| **16 MV** |  |  |  |  |  |  |  |
| TB1B | 0.015 | 0.00 | 0.123 | – 0.066 | 0.026 | 0.144 | – 0.043 |
| TB2B | 0.014 | 0.05 | 0.182 | – 0.007 | 0.020 | 0.146 | – 0.041 |
| *ELM DLG model* | *0.015* |  |  | *– 0.036* |  |  |  |
